# *CASP7* variants modify susceptibility to cervical cancer in Chinese women

**DOI:** 10.1038/srep09225

**Published:** 2015-03-18

**Authors:** Ting-Yan Shi, Jing He, Meng-Yun Wang, Mei-Ling Zhu, Ke-Da Yu, Zhi-Ming Shao, Meng-Hong Sun, Xiaohua Wu, Xi Cheng, Qingyi Wei

**Affiliations:** 1 Cancer Institute, Fudan University Shanghai Cancer Center, Shanghai 200032, China; 2 Department of Obstetrics and Gynecology, Zhongshan Hospital Fudan University, Shanghai 200032, China; 3 State Key Laboratory of Oncology in South China, Department of Experimental Research, Collaborative Innovation Center for Cancer Medicine, Sun Yat-Sen University Cancer Center, Guangzhou, Guangdong 510060, China; 4 Department of Breast Surgery, Fudan University Shanghai Cancer Center, Shanghai 200032, China; 5 Department of Pathology, Fudan University Shanghai Cancer Center, Shanghai 200032, China; 6 Department of Gynecologic Oncology, Fudan University Shanghai Cancer Center, Shanghai 200032, China; 7 Duke Cancer Institute, Duke University Medical Center, Durham, NC 27710, USA

## Abstract

Polymorphisms in *Caspase-7* (*CASP7*) may modulate the programmed cell death and thus contribute to cervical cancer risk. In this case-control study of 1,486 cervical cancer cases and 1,301 controls, we investigated associations between four potentially functional polymorphisms in *CASP7* and cervical cancer risk and evaluated their locus-locus interaction effects on the risk. The genotype-phenotype correlation was performed by a generalized linear regression model. We found that the rs4353229 polymorphism was associated with cervical cancer risk (under a recessive model: crude OR = 1.20, 95% CI = 1.02–1.40). Compared with the TT genotype, the rs10787498GT genotype was associated with an increased cervical cancer risk (adjusted OR = 1.19, 95% CI = 1.00–1.41). Combination analysis showed that subjects with four putative risk genotypes had a 1.54-fold increased cancer risk, compared with those who carried three or less putative risk genotypes. We also observed significant locus-locus joint effects on the risk, which may be mediated by the polymorphisms regulating *CASP7* mRNA expression. Subsequent multifactor dimensionality reduction and classification and regression tree analyses indicated that the *CASP7* genotypes might have a locus-locus interaction effect that modulated cervical cancer risk. Out data suggest that *CASP7* polymorphisms may interact to modify cervical cancer risk by a possible mechanism of regulating *CASP7* mRNA expression.

Cervical cancer is one of the leading cancers in women worldwide, with 529,800 new-diagnosed cancer cases and 275,100 cancer deaths in 2008^1^. More than 85% of these cases and deaths occur in developing countries, including China^1^. Accumulated molecular epidemiologic data support the hypothesis that persistent infection with oncogenic high-risk types of human papillomavirus (HPV) is the primary, even necessary cause of cervical cancer^1^,^2^. However, only a small fraction of women with HPV infection eventually develop cervical cancer, suggesting a wide range of inter-individual genetic variability in cervical cancer susceptibility^3^. Recently, two genome-wide association studies showed that some single nucleotide polymorphisms (SNPs) in the major histocompatibility complex region were associated with cervical cancer risk in both Caucasian and Chinese Han populations^4^,^5^. Despite these successes in identifying genetic variants for cervical cancer risk, the causal variants and/or mechanisms underlying the etiology have been determined for only a small fraction of these associations^6^. Recently, investigations of potentially functional SNPs have now been increasingly advocated across diseases. For example, SNPs at microRNA (miRNA)-binding sites in the 3′-untranslated region (UTR) can remarkably alter the biogenesis and/or function of the corresponding miRNAs and thus contribute to cervical carcinogenesis^7^.

Caspases, at the heart of the apoptotic machinery, encode an evolutionary conserved family of cysteine-aspartic acid proteases and coordinate in cellular regulation and execution of apoptosis^8^. Together with caspase-3 and -6, caspase-7 belongs to the subgroup of executioner caspases^9^, and it executes a coordinated program of proteolysis that leads to the final programmed cell death^10^. Besides its activation during apoptosis, proteolytic maturation of caspase-7 has also been observed in inflammatory conditions^11^, which indicates a potential mechanism of caspase-7 involving the process of HPV infection and host immune response in cervical cancer. Previous genetic association studies had revealed that polymorphisms in the *Caspase-7* (*CASP7*) gene may modulate the default programmed cell death, thus leading to genomic instability and contributing to inter-individual variation in cancer susceptibility^12^,^13^.

To date, no published studies have investigated associations between functional *CASP7* SNPs and cervical cancer risk, besides genome-wide association studies. Herein, we performed a relatively large case-control study to test the hypothesis that potentially functional SNPs in the *CASP7* 3′-UTR are independently and/or jointly associated with cervical cancer risk.

## Results

### Population characteristics

The selected characteristics of the study subjects are listed in Supplementary Table S1. There was no significant difference in distributions of age between the 1,486 cases and 1,301 controls (*P* = 0.126) as result of matching. However, the differences in age at primiparity, menopausal status and body mass index (BMI) were significant between cases and controls. Therefore, we subsequently adjusted these variables for any residual confounding effect in multivariate logistic regression analyses.

### Association of *CASP7* SNPs with cervical cancer risk

As shown in Table 1, compared with CC/CT genotypes, the rs4353229TT genotype was associated with a significantly increased risk of cervical cancer [crude odds ratio (OR) = 1.20, 95% confidence interval (CI) = 1.02–1.40], but after adjustment for age, age at primiparity, menopausal status and BMI, this association was no longer statistically significant. In addition, the rs10787498GT genotype was associated with an increased risk of cervical cancer, compared with the TT genotype (adjusted OR = 1.19, 95% CI = 1.00–1.41). No risk association was observed for the other two SNPs (i.e., rs12247479 and rs1127687), nor for the haplotypes of these four *CASP7* SNPs (Supplementary Table S2). However, when combining these four SNPs and assuming a dominant genetic model, we found that those women who carried four putative risk genotypes had a 1.54-fold increased risk (95% CI = 1.07–2.22) of cervical cancer, compared with those who carried three or less putative risk genotypes (Table 1). Further stratified analyses showed that the significantly increased risk of cervical cancer associated with the rs10787498 GT/GG genotype was more prominent in women younger at primiparity (adjusted OR = 1.40, 95% CI = 1.08–1.82, *P* for homogeneity test = 0.014; Supplementary Table S3).

In further logistic regression analyses, we observed a significant locus-locus multiplicative interaction between rs1127687 and rs12247479 as well as between rs1127687 and rs10787498 (*P* = 0.016 and 0.007, respectively; data not shown). We then explored their two-locus joint effects. As shown in Table 2, women who carried rs1127687AG/AA-rs12247479AG/AA genotypes and those who carried rs1127687AG/AA-rs10787498GT/GG genotypes had a significantly increased risk of cervical cancer, compared with carriers of rs1127687GG-rs12247479GG and rs1127687GG-rs10787498TT, respectively (adjusted OR = 1.48 and 1.52, 95% CI = 1.02–2.15 and 1.13–2.06, *P* for homogeneity = 0.016 and 0.007; respectively). Although no difference in risk estimates by the homogeneity tests, we observed a highly associated risk for the presence of rs10787498GT/GG and rs4353229TT genotypes (adjusted OR = 1.27, 95% CI = 1.04–1.55) as well as a board-line significance for the joint effect of rs12247479AG/AA with rs4353229TT genotypes and for that of rs10787498G with/without rs12247479A allele (adjusted OR = 1.23 and 1.15, 95% CI = 0.97–1.55 and 0.98–1.34; respectively).

We then calculated false-positive report probability (FPRP) values for all observed significant associations (Table 3). The rs4353229TT genotype was associated with an increased risk of cervical cancer with a statistical power of 99.8%, compared with CT/CC genotypes. When the assumption of prior probability was 0.1, the association with rs4353229 was noteworthy in all patients and in the subgroup of postmenopausal women (FPRP = 0.190 and 0.191, respectively), similar for the association of rs10787498GT/GG genotypes in the subgroup of younger at primiparity (FPRP = 0.043) as well as for that of the four putative risk genotype combination effect (FPRP = 0.162). Meanwhile, the two-locus joint effect was still noteworthy for rs10787498-rs4353229 and rs1127687-rs10787498 (FPRP = 0.020 and 0.024, respectively).

### Association of high-order interactions with cervical cancer risk

**Figure 1:**
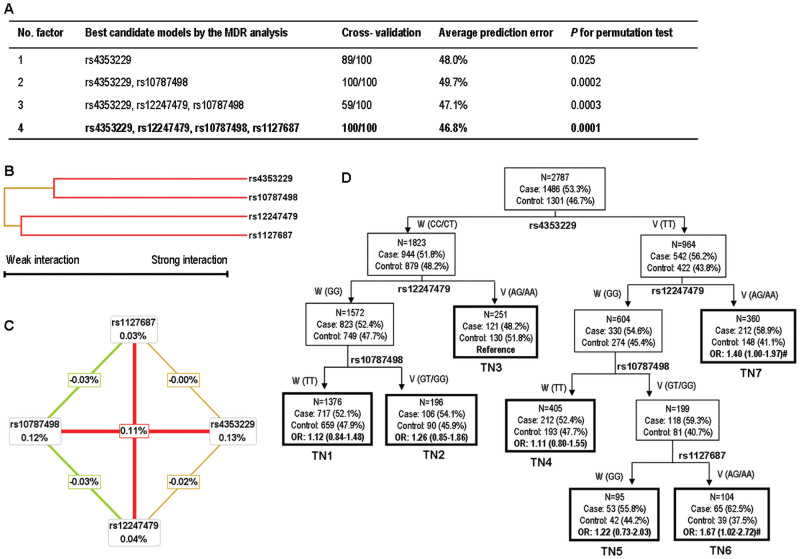
High-order interaction analyses for the four *CASP7* SNPs. (A) The best multifactor dimensionality reduction interaction models. The multi-locus model with maximum cross-validation consistency and minimum prediction error rate is indicated in bold. (B) Interaction dendrogram. The color indicates the strength of the dependence: green is weak and red is strong. (C) Interaction entropy graph. Each SNP is shown in a box with the percent of entropy (main effect). Two-way interactions between SNPs are depicted as an arrow accompanied by a percent of entropy (interaction effect). In the interaction graph, rs4353229 alone eliminates 0.13% of class entropy and has the largest univariate effect. Only small percentages of entropy were explained by rs12247479 (0.04%) or rs1127687 (0.03%) when considered independently, while a large percentage of entropy was explained by their pairwise interactions (0.11%), indicating a synergistic interaction. (D) Classification and regression tree. Terminal nodes are thick bordered. W, wild type genotype; V, variant genotype; TN, terminal node; #, *P* value <0.05.

We further performed the multifactor dimensionality reduction (MDR) analysis and found that rs4353229 was the best one-factor model with the highest cross-validation consistency (CVC) (89%) and the lowest prediction error (48.0%) among all four SNPs. Additionally, the four-locus model had a maximal CVC (100%) and a minimal prediction error (46.8%), suggesting a better prediction than other models (Figure 1A). Subsequent hierarchical cluster analysis placed rs4353229 and rs10787498, rs12247479 and rs1127687 on the same branch (Figure 1B), suggesting that this four-locus model might have an interaction effect by modulating cervical cancer risk, which is also supported by the interaction graph (Figure 1C). Moreover, consistent with the findings in the single locus analysis, rs4353229 (0.13%) and rs10787498 (0.12%) showed a strong effect on cervical cancer risk (Figure 1C).

By the classification and regression tree (CART) analysis, we found rs4353229 to be the initial split of root nodes, indicating that rs4353229 was the strongest risk factor for cervical cancer among these four SNPs. Further inspection of the tree structure revealed distinct interaction patterns. Women carrying rs4353229TT, rs12247479GG, rs10787498GT/GG and rs1127687AG/AA genotypes [terminal node (TN) 6] had a 1.67-fold increased risk of cervical cancer, compared with the TN3 group at the lowest risk (*P* = 0.041; Figure 1D).

### Correlation between *CASP7* genotypes and mRNA expression levels

**Figure 2:**
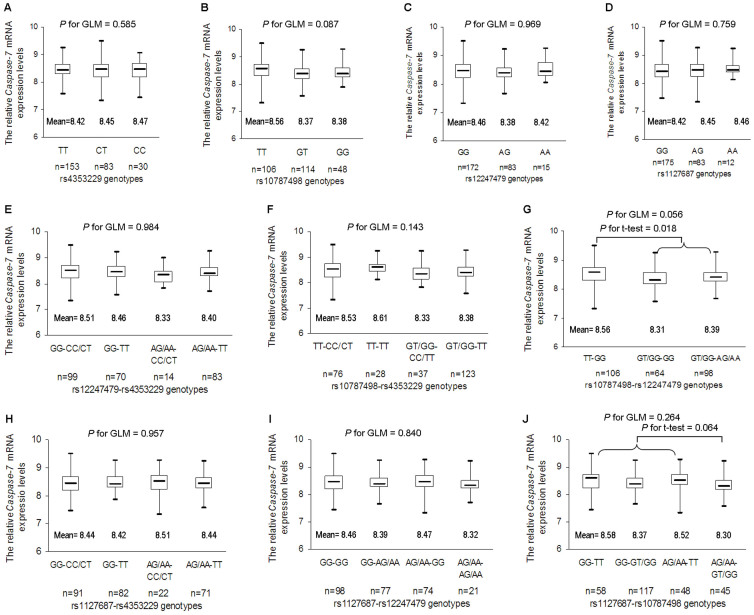
The relative expression levels of *CASP7* mRNA by different genotypes in 270 HapMap subjects. (A) rs4353229, (B) rs10787498, (C) rs12247479, (D) rs1127687, as well as the joint effects of (E) rs12247479 with rs4353229, (F) rs10787498 with rs4353229, (G) rs10787498 with rs12247479, (H) rs1127687 with rs4353229, (I) rs1127687 with rs12247479 and (J) rs1127687 with rs10787498 are evaluated by generalized linear models and Student's *t* tests.

In 270 HapMap individuals whose mRNA expression data were available, although there was no correlation of *CASP7* mRNA expression levels with the risk loci, we did observe a board-line significant correlation of *CASP7* mRNA expression levels with the joint effect of rs10787498 and rs12247479 [generalized linear model (GLM), *P* = 0.056; Figure 2G]. Moreover, *CASP7* mRNA expression levels showed an increased trend for rs10787498TT-rs12247479GG carriers and a decreased trend for rs1127687AG/AA-rs10787498GT/GG carriers (Student's *t* test, *P* = 0.018 and 0.064, respectively; Figure 2G, 2J).

## Discussion

In this case-control study of 1,486 cervical cancer cases and 1,301 female controls, we found that the rs4353229TT genotype was associated with an increased risk of cervical cancer with a statistical power of 99.8%. Moreover, we also observed significant joint effects and locus-locus interactions of the *CASP7* SNPs on cervical cancer risk. This is, to the best of our knowledge, the first report that describes the associations between potentially functional SNPs in *CASP7* and cervical cancer risk. Our study is also among the few that have examined the locus-locus interaction in the etiology of cervical cancer.

*CASP7*, located at chromosome 10q25, encodes a member of cysteine peptidase and has been identified as one of the three downstream effectors in the apoptosis pathway in mammalian cells^9^, involved in the execution-phase process of cellular apoptosis. Previous data demonstrated that genetic variations in apoptosis genes might modulate the programmed cell death in various biological systems and alter tissue response to irradiation and cytotoxic chemotherapy^14^, thus eventually leading to genomic instability and tumorigenesis in humans^15^. It is of note that the resistance to apoptosis is an important indicator related to cervical carcinogenesis^16^. In cervical cancer cells, the lack of caspase-mediated apoptosis due to unresponsiveness to pro-apoptotic stimuli causes uncontrolled cell proliferation^17^.

Recently, Wang et al. reported that the rs4353229TT genotype was associated with 0.83-fold decreased risk of gastric cancer^13^. Inversely, in the current study, we found a possibly increased risk of cervical cancer for the rs4353229TT genotype. This discrepancy might be partly due to tumor specificity and population stratification. On the other hand, we also observed that this risk association might be modified by environmental variables and that the effect of one single *CASP7* locus on cervical cancer risk might be weak. Indeed, for cancer biology, the functional characterization of risk loci as well as the complex interplay among multiple loci in many cancers poses a particular exciting challenge for the era of post genome-wide association study.

In the present study, we did find that potentially functional SNPs at *CASP7* 3′-UTR might be jointly associated with cervical cancer risk. Further genotype-phenotype analyses suggested an association of *CASP7* mRNA expression levels with the joint effect between rs10787498 and rs12247479 as well as between rs1127687 and rs10787498. Consistently, the locus-locus joint effect association analyses demonstrated that there was a super-multiplicative joint effect between rs1127687 and rs10787498 as well as possibly between rs10787498 and rs12247479 on cervical cancer risk. These findings indicated that *CASP7* SNPs might interact to modify cervical cancer risk by affecting *CASP7* mRNA expression. Subsequent high-order interaction analyses also helped to explain this paradigm. The best interaction model revealed that the four *CASP7* SNPs interacted with a maximal CVC and a minimal prediction error, which was more evident in the interaction entropy analysis. Additionally, the CART analysis identified subsets of individuals with cervical cancer risk based on various combinations of genotypes, and the OR for individuals in each TN ranged from 1.11 to 1.67, which also suggests a synergistic interaction between these four SNPs.

Despite the strengths and biologic plausibility of the associations observed in the current study, several limitations need to be addressed. Firstly, there may be selection and information bias originated from a retrospective study design, which may have been minimized by frequency-matching for cases and controls as well as the adjustment for potential confounding factors in multivariate analyses. Secondly, the *P* value of Hardy-Weinberg equilibrium (HWE) was 0.034 for rs10787498, but given that the deviation from HWE among controls was defined as a significance level of α <10^−3^ or 10^−4^, all the SNPs in our analyses were in agreement with HWE. Finally, because the lack of routine HPV screening for all cases and controls in our hospital, we could not evaluate HPV infection as the potential confounder in risk estimates of cervical cancer.

In summary, in the current case-control study of 1,486 cases and 1,301 controls, we found that *CASP7* SNPs might be associated with cervical cancer risk in Eastern Chinese women. There were substantial joint effects and locus-locus interactions among these SNPs, and such effects may contribute to cervical cancer risk by affecting *CASP7* mRNA expression. However, well-designed, larger, and prospective studies with detailed information about HPV infection are warranted to validate our findings.

## Methods

### Study subjects

The recruitment of the cases and controls was partly described previously^7^. Briefly, all subjects were unrelated ethnic Han Chinese and residents in the Eastern China. The 1,486 newly diagnosed and histopathologically confirmed primary cervical cancer patients were consecutively recruited and collected by the tissue bank of Fudan University Shanghai Cancer Center (FUSCC). The 1,301 frequency-matched healthy controls without history of cancers were recruited from women who had come to FUSCC for breast cancer screening. After a written informed consent was obtained, all subjects were interviewed to collect their demographic and risk factor information. Because most Chinese women did not smoke cigarettes or drink alcohol, all participants included in the analysis were non-smokers and non-drinkers, and provided a one-time 10 mL of venous blood sample (after diagnosis and before the initiation of treatment for cases). The experimental and research protocols were approved by the Institutional Review Board of FUSCC, and all experiment on humans was performed in accordance with relevant guidelines and regulations.

### SNP selection and genotyping

By searching the NCBI dbSNP database (http://www.ncbi.nlm.nih.gov/projects/SNP) and the International HapMap Project database (http://hapmap.ncbi.nlm.nih.gov/), we found that there were 22 SNPs in *CASP7* 3′-UTR, of which four were finally selected for genotyping, based on the following criteria: 1) minor allele frequency of at least 5% in Chinese populations, 2) with low linkage disequilibrium by using an *r*^2^ threshold of <0.8 for each other, 3) predicted to be potentially functional by the SNP function prediction platform (http://snpinfo.niehs.nih.gov/snpinfo/snpfunc.htm), and 4) not included and published in genome-wide association studies. Thus, the selected SNPs were rs4353229 T> C, rs12247479 G> A, rs10787498 T> G and rs1127687 G> A. Genomic DNA extraction and genotyping were conducted as described previously^18^. As a result, the discrepancy rate in all positive controls (i.e., duplicated samples, overlapping samples from previous studies and samples randomly selected to be sequenced) was less than 0.1%.

### Genotype-phenotype correlation analysis

To evaluate biological plausibility of our findings, we used the data on *CASP7* genotypes and *CASP7* mRNA expression levels both available for 270 HapMap subjects by SNPexp online tool (http://app3.titan.uio.no/biotools/help.php?app=snpexp) and conducted genotype-phenotype correlation analysis as described previously^18^,^19^.

### Statistical analysis

HWE was tested by χ^2^-test for each SNP. We performed the Pearson's χ^2^-test for the differences in selected variables between cases and controls. The association of *CASP7* genotypes with cervical cancer risk was estimated by computing ORs and their 95% CIs from both univariate and multivariate logistic regression models. We also evaluated the associations in subgroup and joint effect analyses. The PROC HAPLOTYPE procedure in SAS software was applied to infer haplotype frequencies among the four SNPs. To avoid false positive associations in this study, we calculated the FPRP with the assumption of different prior probabilities (0.0001, 0.001, 0.01, 0.1 and 0.25). FPRP values <0.2 were considered to be noteworthy^20^. We used GLM for the genotype-phenotype correlation, and used student's *t* test and analysis of variance test to evaluate the differences in the relative mRNA expression levels among different genotype groups.

The MDR and CART analyses were conducted by the MDR V2.0 beta 8.2 program (http://www.multifactordimensionalityreduction.org/) and SAS software (version 9.1; SAS Institute, Cary, NC), respectively, as described previously^21^. Briefly, we enrolled the four risk loci in the MDR analysis to identify the best *n*-factor interaction model. Then, we performed the interaction dendrograms and graphs^22^. The color of branches and lines is referred to the type of interaction, green-to-yellow-to-red indicates a weak-to-strong interaction. CART creates a decision tree that depicts how well each genotype predicts disease and ends up with TNs.

All statistical analyses were performed with SAS 9.1 software (SAS Institute, Cary, NC), unless stated otherwise. All *P* values were two-sided with a significance level of *P* <0.05.

## Author Contributions

All authors contributed significantly to this work. Conceived and designed the study strategy: X.C. & Q.W. Designed the experiment: Q.W. & T.-Y.S. Recruited the participants and collected their information and blood samples: T.-Y.S., J.H., K.-D.Y., Z.-M.S., M.-H.S. & X.W. Performed the experiments: T.-Y.S., M.-Y.W. & M.-L.Z. Statistical analyses: T.-Y.S. & J.H. Wrote the manuscript: T.-Y.S. & Q.W. All authors reviewed the manuscript. In addition, all authors approved the final draft.

## Supplementary Material

Supplementary Information Supplementary Information

## Figures and Tables

**Table 1:** Associations of *CASP7* genotypes with the risk of cervical cancer

Variants Genotypes	Cases (N = 1,486)	Controls (N = 1,301)	*P*^a^	Crude OR (95% CI)	*P*	Adjusted OR (95%CI)^a^	*P*^b^
***CASP7-*rs4353229**	**HWE = 0.566**						
CC	236 (15.9)	226 (17.4)		1.00		1.00	
CT	708 (47.6)	653 (50.2)		1.04 (0.84–1.28)	0.727	1.05 (0.84–1.31)	0.687
TT	542 (36.5)	422 (32.4)		1.23 (0.99–1.54)	0.068	1.19 (0.94–1.50)	0.154
Additive model			0.077	1.12 (1.01–1.25)	**0.036**	1.10 (0.98–1.23)	0.110
Dominant model			0.291	1.11 (0.91–1.36)	0.291	1.10 (0.89–1.36)	0.367
Recessive model			**0.025**	1.20 (1.02–1.40)	**0.026**	1.15 (0.97–1.35)	0.106
***CASP7-*rs12247479**	**HWE = 0.298**						
GG	1,153 (77.6)	1,023 (78.6)		1.00		1.00	
AG	315 (21.2)	257 (19.8)		1.09 (0.90–1.31)	0.375	1.08 (0.89–1.31)	0.431
AA	18 (1.2)	21 (1.6)		0.76 (0.40–1.43)	0.397	0.69 (0.35–1.39)	0.301
Additive model			0.448	1.03 (0.88–1.22)	0.712	1.02 (0.86–1.21)	0.830
Dominant model			0.508	1.06 (0.89–1.27)	0.508	1.05 (0.87–1.27)	0.593
Recessive model			0.366	0.75 (0.40–1.41)	0.367	0.68 (0.34–1.36)	0.279
***CASP7-*rs10787498**	**HWE = 0.034**						
TT	929 (62.5)	857 (65.9)		1.00		1.00	
GT	493 (33.2)	383 (29.4)		1.19 (1.01–1.40)	**0.038**	1.19 (1.00–1.41)	**0.045**
GG	64 (4.3)	61 (4.7)		0.97 (0.67–1.39)	0.860	0.91 (0.62–1.34)	0.637
Additive model			0.104	1.09 (0.96–1.25)	0.173	1.08 (0.94–1.24)	0.257
Dominant model			0.066	1.16 (0.99–1.35)	0.066	1.15 (0.98–1.36)	0.090
Recessive model			0.627	0.92 (0.64–1.31)	0.626	0.86 (0.59–1.26)	0.438
***CASP7-*rs1127687**	**HWE = 0.278**						
GG	896 (60.3)	804 (61.8)		1.00		1.00	
AG	518 (34.9)	429 (33.0)		1.08 (0.92–1.27)	0.325	1.08 (0.91–1.28)	0.371
AA	72 (4.9)	68 (5.2)		0.95 (0.67–1.34)	0.771	0.89 (0.61–1.28)	0.512
Additive model			0.554	1.03 (0.91–1.17)	0.616	1.02 (0.89–1.16)	0.829
Dominant model			0.417	1.07 (0.91–1.24)	0.418	1.05 (0.90–1.24)	0.533
Recessive model			0.646	0.92 (0.66–1.30)	0.645	0.86 (0.60–1.24)	0.416
**Combined effects of four putative risk genotypes by the dominant genetic model**							
0	235 (15.8)	223 (17.1)	0.078	1.00		1.00	
1	264 (17.8)	227 (17.5)		1.10 (0.86–1.42)	0.449	1.09 (0.83–1.42)	0.541
2	587 (39.5)	537 (41.3)		1.04 (0.84–1.29)	0.741	1.03 (0.82–1.30)	0.784
3	308 (20.7)	263 (20.2)		1.11 (0.87–1.42)	0.401	1.10 (0.85–1.42)	0.478
4	92 (6.2)	51 (3.9)		1.71 (1.16–2.52)	**0.007**	1.62 (1.08–2.43)	**0.020**
				*P*_trend_ = 0.076		*P*_trend_^a^ = 0.127	
0–3	1,394 (93.8)	1,250 (96.1)	**0.007**	1.00		1.00	
4	92 (6.2)	51 (3.9)		1.62 (1.14–2.30)	**0.007**	1.54 (1.07–2.22)	**0.021**

^a^χ^2^ test for genotype distributions between cases and controls;

^b^Adjusted for age, age at primiparity, menopausal status, BMI in logistic regress models.

The result were in bold, if *P* <0.05.

**Table 2:** Locus-locus joint effects in associations between *CASP7* genotypes and cervical cancer risk

Locus-locus joint effect		Genotype		Cases	Controls	OR (95% CI)	*P*	OR (95% CI)^a^	*P*^a^	*P*hom
rs12247479	rs4353229	GG	CC/CT	823 (55.4)	749 (57.6)	1.00		1.00		0.076
			TT	330 (22.2)	274 (21.1)	1.10 (0.91–1.32)	0.340	1.07 (0.87–1.30)	0.532	
		AG/AA	CC/CT	121 (8.1)	130 (10.0)	0.85 (0.65–1.11)	0.223	0.88 (0.67–1.16)	0.374	
			TT	212 (14.3)	148 (11.4)	1.30 (1.03–1.64)	**0.028**	1.24 (0.97–1.58)	0.089#	
		AG/AA-TT *vs.* others				1.29 (1.03–1.62)	**0.026**	1.23 (0.97–1.55)	0.091#	
rs10787498	rs4353229	TT	CC/CT	717 (48.3)	663 (51.0)	1.00		1.00		0.070
			TT	212 (14.3)	194 (14.9)	1.01 (0.81–1.26)	0.927	1.00 (0.79–1.26)	0.975	
		GT/GG	CC/CT	227 (15.3)	216 (16.6)	0.97 (0.78–1.20)	0.793	1.01 (0.81–1.27)	0.902	
			TT	330 (22.2)	228 (17.5)	1.33 (1.09–1.63)	**0.005**	1.28 (1.04–1.58)	**0.021**	
		GT/GG-TT *vs.* others				1.34 (1.11–1.62)	**0.002**	1.27 (1.04–1.55)	**0.017**	
	rs12247479	TT	GG	929 (62.5)	852 (65.5)	1.00		1.00		0.102
			AG/AA	0	5 (0.4)	——		——		
		GT/GG	GG	224 (15.1)	171 (13.1)	1.21 (0.97–1.51)	0.091#	1.21 (0.97–1.50)	0.095#	
			AG/AA	333 (22.4)	273 (21.0)	1.12 (0.93–1.35)	0.223	1.12 (0.93–1.34)	0.248	
		1–2 putative risk genotypes *vs.* 0 risk genotype				1.16 (0.99–1.35)	0.065#	1.15 (0.98–1.34)	0.078#	
rs1127687	rs4353229	GG	CC/CT	675 (45.4)	629 (48.4)	1.00		1.00		0.877
			TT	221 (14.9)	175 (13.5)	1.18 (0.94–1.48)	0.158	1.11 (0.87–1.41)	0.397	
		AG/AA	CC/CT	269 (18.1)	250 (19.2)	1.00 (0.82–1.23)	0.980	0.99 (0.80–1.23)	0.911	
			TT	321 (21.6)	247 (19.0)	1.21 (0.99–1.47)	0.063#	1.17 (0.95–1.44)	0.149	
		AG/AA-TT *vs.* others				1.17 (0.97–1.41)	0.094#	1.14 (0.94–1.39)	0.178	
	rs12247479	GG	GG	655 (44.1)	578 (44.4)	1.00		1.00		**0.016**
			AG/AA	241 (16.2)	226 (17.4)	0.94 (0.76–1.17)	0.576	0.94 (0.75–1.17)	0.596	
		AG/AA	GG	498 (33.5)	445 (34.2)	0.99 (0.83–1.17)	0.885	0.98 (0.82–1.17)	0.833	
			AG/AA	92 (6.2)	52 (4.0)	1.56 (1.09–2.23)	**0.015**	1.48 (1.02–2.15)	**0.040**	
		AG/AA-AG/AA *vs.* others				1.57 (1.11–2.22)	**0.012**	1.49 (1.03–2.14)	**0.033**	
	rs10787498	GG	TT	498 (33.5)	451 (34.7)	1.00		1.00		**0.007**
			GT/GG	398 (26.8)	353 (27.1)	1.02 (0.84–1.24)	0.831	1.03 (0.84–1.26)	0.795	
		AG/AA	TT	431 (29.0)	406 (31.2)	0.96 (0.80–1.16)	0.678	0.96 (0.79–1.17)	0.680	
			GT/GG	159 (10.7)	91 (7.0)	1.58 (1.19–2.11)	**0.002**	1.52 (1.13–2.06)	**0.006**	
		AG/AA-GT/GG *vs.* others				1.58 (1.21–2.07)	**0.001**	1.52 (1.15–2.01)	**0.004**	

OR, odds ratio; CI, confidence interval

^a^Obtained in logistic regression models with adjustment for age, age at primiparity, menopausal status, BMI

^hom^Homogeneity test

^#^Boardline significance

The results were in bold, if *P* <0.05

**Table 3:** False-positive report probability values for associations between *CASP7* genotypes and cervical cancer risk

Genotypes	Positive OR (95% CI)*	P*	Statistical power**	Prior probability				
				0.25	0.1	0.01	0.001	0.0001
**rs4353229**								
TT *vs.* CT/CC								
All patients	1.20 (1.02–1.40)	0.026	0.998	**0.072**	**0.190**	0.721	0.963	0.996
Postmenopausal	1.39 (1.06–1.82)	0.019	0.722	**0.073**	**0.191**	0.723	0.963	0.996
**rs10787498**								
GT *vs.* TT								
All patients	1.19 (1.01–1.40)	0.038	0.999	**0.102**	0.255	0.790	0.974	0.997
GT/GG *vs.* TT								
Age at primiparity ≤ 24	1.45 (1.13–1.85)	0.003	0.608	**0.015**	**0.043**	0.328	0.831	0.980
Tumor size <4 cm	1.21 (1.02–1.44)	0.033	0.993	**0.091**	0.230	0.767	0.971	0.997
**rs1127687**								
AG/AA *vs.* GG								
FIGO stage II	1.26 (1.02–1.55)	0.031	0.952	**0.089**	0.227	0.763	0.970	0.997
**Combined effect of putative risk genotypes**								
4 *vs.* ≤ 3	1.62 (1.14–2.30)	0.007	0.326	**0.060**	**0.162**	0.680	0.955	0.995
**rs10787498 - rs4353229 joint effect**								
GT/GG-TT *vs.* TT-CC/CT	1.33 (1.09–1.63)	0.005	0.983	**0.017**	**0.048**	0.357	0.848	0.982
GT/GG-TT *vs.* others	1.34 (1.11–1.62)	0.002	0.888	**0.007**	**0.020**	**0.182**	0.692	0.957
**rs1127687 - rs12247479 joint effect**								
AG/AA-AG/AA *vs.* GG-GG	1.56 (1.09–2.23)	0.015	0.414	**0.098**	0.246	0.782	0.973	0.997
AG/AA-AG/AA *vs.* others	1.57 (1.11–2.22)	0.012	0.404	**0.082**	0.211	0.746	0.967	0.997
**rs1127687 - rs10787498 joint effect**								
AG/AA-GT/GG *vs.* GG-TT	1.58 (1.19–2.11)	**0.002**	0.369	**0.016**	**0.047**	0.349	0.844	0.982
AG/AA-GT/GG *vs.* others	1.58 (1.21–2.07)	**0.001**	0.362	**0.008**	**0.024**	0.215	0.734	0.965

OR, odds ratio; CI, confidence interval; SCC, squamous cell carcinoma; FIGO, International Federation of Gynecology and Obstetrics; LN, Lymph Node; LVSI, lymph-vascular space invasion; ER, estrogen receptor; PR, progesterone receptor.

*Crude OR and *P* value;

**Statistical power was calculated using the number of observations in the subgroup and the ORs and *P* values in this table.

The results in false-positive report probability analysis were in bold, if the prior probability < 0.20.
