# Supplementary material for: CASP7 variants modify susceptibility to cervical cancer in Chinese women
Source: Sci Rep. 2015 Mar 18;5:9225. doi: 10.1038/srep09225 (PMC4363885; doi:10.1038/srep09225)
Supplement: Supplementary Information [file srep09225-s1.doc]

**Supplemental Tables:**

***CASP7* variants modify susceptibility to cervical cancer in Chinese women**

Ting-Yan Shi ^1,2^, Jing He ^2,3^, Meng-Yun Wang ^2^, Mei-Ling Zhu ^2^, Ke-Da Yu ^4^, Zhi-Ming Shao ^4^, Meng-Hong Sun ^5^, Xiaohua Wu ^6^, Xi Cheng ^6,*^, Qingyi Wei ^2,7,*^

1 Department of Obstetrics and Gynecology, Zhongshan Hospital Fudan University, Shanghai 200032, China

2 Cancer Institute, Fudan University Shanghai Cancer Center, Shanghai 200032, China

3 State Key Laboratory of Oncology in South China, Department of Experimental Research, Collaborative Innovation Center for Cancer Medicine, Sun Yat-Sen University Cancer Center, Guangzhou, Guangdong 510060, China

4 Department of Breast Surgery, Fudan University Shanghai Cancer Center, Shanghai 200032, China

5 Department of Pathology, Fudan University Shanghai Cancer Center, Shanghai 200032, China

6 Department of Gynecologic Oncology, Fudan University Shanghai Cancer Center, Shanghai 200032, China

7 Duke Cancer Institute, Duke University Medical Center, Durham, NC 27710, USA

***Correspondence to：**Xi Cheng, MD, PhD, Professor of Gynecologic Oncology, Department of Gynecologic Oncology, Fudan University Shanghai Cancer Center; 270 Dongan Road, Shanghai 200032, China. Tel: (+86-21) 64175590; Fax: (+86-21) 64174774; E-mail: [cheng_xi1@hotmail.com](mailto:cheng_xi1@hotmail.com), or Qingyi Wei, MD, PhD, Cancer Institute, Fudan University Shanghai Cancer Center, Shanghai, China. Tel: (+86-21) 64175590-5315, Fax: (+86-21) 64172585; E-mail: [weiqingyi@yahoo.com](mailto:weiqingyi@yahoo.com) or Duke Cancer Institute, Duke University Medical Center, Durham, NC 27710, USA; E-mail: [qingyi.wei@duke.edu](mailto:qingyi.wei@duke.edu).

| **Supplementary Table S1.** Distributions of selected variables in cervical cancer cases and cancer-free controls | | | | |
| --- | --- | --- | --- | --- |
|
| **Variables** | **Cases N (%)** | **Controls N (%)** | ***P*^a^** |  |
| All subjects | 1,486 (100) | 1,301 (100) |  |  |
| Age, yr (Mean±SD) | 45.9 ± 9.8 | 46.6 ± 8.8 | 0.126 |  |
| < 46 (mean) | 757 (50.9) | 625 (48.0) |  |  |
| ≥ 46 (mean) | 729 (49.1) | 676 (52.0) |  |  |
| Age of primiparity, yr (Mean±SD) | 23.5 ± 2.9 | 24.4 ± 2.4 | <.0001 |  |
| ≤ 24 (mean) | 887 (63.4) | 665 (51.8) |  |  |
| > 24 (mean) | 512 (36.6) | 618 (48.2) |  |  |
| Missing | 87 | 18 |  |  |
| Menopausal status |  |  | <.0001 |  |
| Premenopausal | 1,067 (72.4) | 756 (58.2) |  |  |
| Postmenopausal | 407 (27.6) | 543 (41.8) |  |  |
| Missing | 12 | 2 |  |  |
| BMI^b^, kg/m^2^ |  |  | <.0001 |  |
| < 25 | 1,140 (78.1) | 847 (65.2) |  |  |
| ≥ 25 | 319 (21.9) | 453 (34.9) |  |  |
| Missing | 27 | 1 |  |  |
| Histology ^b^ |  |  |  |  |
| CIN-III | 160 (10.8) |  |  |  |
| SCC | 1,164 (78.6) |  |  |  |
| Adenocarcinoma | 102 (6.9) |  |  |  |
| Adenosquamous | 37 (2.5) |  |  |  |
| Others | 18 (1.2) |  |  |  |
| Missing | 5 |  |  |  |
| FIGO stage |  |  |  |  |
| I | 691 (55.6) |  |  |  |
| II | 503 (40.5) |  |  |  |
| III | 44 (3.5) |  |  |  |
| IV | 4 (0.3) |  |  |  |
| Missing | 244 |  |  |  |
| Tumor size, cm |  |  |  |  |
| < 4 | 908 (66.8) |  |  |  |
| ≥ 4 | 451 (33.2) |  |  |  |
| Missing | 127 |  |  |  |
| Pelvic LN |  |  |  |  |
| Negative | 1,091 (77.4) |  |  |  |
| Positive | 318 (22.6) |  |  |  |
| Missing | 77 |  |  |  |
| LVSI |  |  |  |  |
| Negative | 839 (67.0) |  |  |  |
| Positive | 413 (33.0) |  |  |  |
| Missing | 234 |  |  |  |
| Depth of cervical stromal invasion | |  |  |  |
| ≤ 1/2 | 665 (47.9) |  |  |  |
| > 1/2 | 723 (52.1) |  |  |  |
| Missing | 98 |  |  |  |
| ER expression | |  |  |  |
| Negative | 745 (92.1) |  |  |  |
| Positive | 64 (7.9) |  |  |  |
| Missing | 677 |  |  |  |
| PR expression | |  |  |  |
| Negative | 783 (96.8) |  |  |  |
| Positive | 26 (3.2) |  |  |  |
| Missing | 677 |  |  |  |
| FIGO, International Federation of Gynecology and Obstetrics; CIN, cervical intraepithelial neoplasia; SCC, squamous cell carcinoma; LN, Lymph Node; LVSI, lymph-vascular space invasion. | | | | |
| ^a^ Two-sided *χ^2^* test for distributions between cases and controls; | | | | |
| ^b^ According to the current WHO recommendations. | | | | |

| **Supplementary Table S2.** Haplotype analysis for genotypes of *CASP7* and cervical cancer risk | | | | | | | | |
| --- | --- | --- | --- | --- | --- | --- | --- | --- |
|  |  |  |  |  |  |  |  |  |
| **Haplotypes** | **Cases** | | **Controls** | | **Crude OR** | ***P*** | **Adjusted OR^a^** | ***P*^a^** |
|  | **(N =2,972)** | | **(N =2,602)** | | **(95% CI)** |  | **(95% CI)** |  |
|  | **N** | **%** | **N** | **%** |  |  |  |  |
| C_rs4353229_ G_rs12247479_  T_10787498_ G_1127687_ | 1,173 | 39.5 | 1,094 | 42.0 | 1.00 |  | 1.00 |  |
| C_rs4353229_ A_rs12247479_  T_10787498_ G_1127687_ | 0 | 0.0 | 3 | 0.1 | 2.11 (0.41-10.95) | 0.375 | 2.09 (0.40-11.00) | 0.383 |
| C_rs4353229_ G_rs12247479_  G_10787498_ G_1127687_ | 5 | 0.2 | 2 | 0.1 | 0.28 (0.06-1.40) | 0.122 | 0.36 (0.07-1.83) | 0.216 |
| C_rs4353229_ G_rs12247479_  T_10787498_ A_1127687_ | 2 | 0.1 | 6 | 0.2 | 0.90 (0.76-1.08) | 0.26 | 0.93 (0.77-1.12) | 0.425 |
| T_rs4353229_ A_rs12247479_  G_10787498_ G_1127687_ | 351 | 11.8 | 293 | 11.3 | —— | 0.962 | —— | 0.951 |
| T_rs4353229_ G_rs12247479_  G_10787498_ A_1127687_ | 0 | 0.0 | 3 | 0.1 | 1.06 (0.84-1.35) | 0.609 | 1.06 (0.83-1.37) | 0.603 |
| T_rs4353229_ G_rs12247479_  G_10787498_ G_1127687_ | 265 | 8.9 | 210 | 8.1 | 1.01 (0.82-1.23) | 0.944 | 1.02 (0.82-1.26) | 0.879 |
| T_rs4353229_ G_rs12247479_  T_10787498_ A_1127687_ | 660 | 22.2 | 559 | 21.5 | —— | —— | —— | —— |
| T_rs4353229_ G_rs12247479_  T_10787498_ G_1127687_ | 516 | 17.4 | 432 | 16.6 | —— | —— | —— | —— |
| CI, confidence interval; OR, odds ratio  ^a^ Obtained in logistic regression models with adjustment for age, age at primiparity, menopausal status, BMI  The results were in bold, if *P* < 0.05 | | | | | | | | |

| **Supplementary Table S3.** Stratification analysis for associations between *CASP7* genotypes and cervical cancer risk in Eastern Chinese women | | | | | | | | | | | | | | | | | | | | |
| --- | --- | --- | --- | --- | --- | --- | --- | --- | --- | --- | --- | --- | --- | --- | --- | --- | --- | --- | --- | --- |
| **Variables** | **rs4353229** | | **Adjusted OR^a^ (95% CI)** | ***P^a^*** | ***P*^hom^** | **rs12247479** | | **Adjusted OR^a^ (95% CI)** | ***P^a^*** | ***P*^hom^** | **rs10787498** | | **Adjusted OR^a^ (95% CI)** | ***P*^a^** | ***P*^hom^** | **rs1127687** | | **Adjusted OR^a^ (95% CI)** | ***P*^a^** | ***P*^hom^** |
|  | **(cases/controls)** | |  |  |  | **(cases/controls)** | |  |  |  | **(cases/controls)** | |  |  |  | **(cases/controls)** | |  |  |  |
|  | **CC/CT** | **TT** |  |  |  | **GG** | **AG/AA** |  |  |  | **TT** | **GT/GG** |  |  |  | **GG** | **AG/AA** |  |  |  |
| Age, yr |  |  |  |  |  |  |  |  |  |  |  |  |  |  |  |  |  |  |  |  |
| ≤46 (mean) | 478/411 | 279/214 | 1.09 (0.87-1.38) | 0.460 | 0.437 | 572/487 | 185/138 | 1.14 (0.86-1.45) | 0.417 | 0.393 | 456/404 | 301/221 | 1.22 (0.97-1.53) | 0.092 | 0.558 | 477/392 | 280/233 | 0.93 (0.74-1.17) | 0.554 | 0.607 |
| >46 (mean) | 466/468 | 263/208 | 1.26 (0.99-1.60) | 0.062 |  | 581/536 | 148/140 | 1.00 (0.76-1.32) | 0.999 |  | 473/453 | 256/223 | 1.10 (0.87-1.39) | 0.442 |  | 419/412 | 310/264 | 1.22 (0.97-1.54) | 0.087 |  |
| Age at primiparity, yr | |  |  |  |  |  |  |  |  |  |  |  |  |  |  |  |  |  |  |  |
| ≤24 (mean) | 564/447 | 328/218 | 1.18 (0.95-1.46) | 0.143 | 0.825 | 690/509 | 197/156 | 0.94 (0.74-1.20) | 0.615 | 0.117 | 562/416 | 325/249 | 0.98 (0.80-1.22) | 0.876 | **0.014** | 523/421 | 364/244 | 1.19 (0.96-1.47) | 0.112 | 0.053 |
| >24 (mean) | 333/419 | 179/199 | 1.11 (0.86-1.44) | 0.425 |  | 394/499 | 118/119 | 1.23 (0.91-1.66) | 0.183 |  | 311/427 | 201/191 | 1.40 (1.08-1.82) | **0.010** |  | 324/372 | 188/246 | 0.91 (0.71-1.17) | 0.464 |  |
| Menopausal status | |  |  |  |  |  |  |  |  |  |  |  |  |  |  |  |  |  |  |  |
| Premenopausal | 682/496 | 385/260 | 1.06 (0.87-1.29) | 0.581 | 0.139 | 825/582 | 242/174 | 0.98 (0.78-1.24) | 0.890 | 0.348 | 664/488 | 403/268 | 1.12 (0.91-1.36) | 0.283 | 0.528 | 664/473 | 403/283 | 0.97 (0.79-1.18) | 0.743 | 0.598 |
| Postmenopausal | 256/381 | 151/162 | 1.37 (1.02-1.84) | **0.035** |  | 318/439 | 89/104 | 1.17 (0.83-1.65) | 0.361 |  | 256/367 | 151/176 | 1.15 (0.86-1.54) | 0.335 |  | 225/330 | 182/213 | 1.30 (0.98-1.73) | 0.067 |  |
| BMI^b^, kg/m^2^ |  |  |  |  |  |  |  |  |  |  |  |  |  |  |  |  |  |  |  |  |
| < 25 | 723/565 | 417/282 | 1.10 (0.91-1.34) | 0.321 | 0.633 | 891/665 | 249/182 | 1.02 (0.82-1.28) | 0.832 | 0.475 | 714/563 | 426/284 | 1.19 (0.98-1.44) | 0.077 | 0.701 | 678/518 | 462/329 | 1.07 (0.88-1.29) | 0.496 | 0.621 |
| ≥ 25 | 204/313 | 115/140 | 1.28 (0.93-1.76) | 0.134 |  | 242/357 | 77/96 | 1.09 (0.76-1.56) | 0.639 |  | 199/293 | 120/160 | 1.04 (0.76-1.42) | 0.804 |  | 202/285 | 117/168 | 1.02 (0.75-1.39) | 0.913 |  |
| Histology^b^ |  |  |  |  |  |  |  |  |  |  |  |  |  |  |  |  |  |  |  |  |
| CINIII | 100/879 | 60/422 | 1.17 (0.81-1.67) | 0.410 | 0.409 | 123/1,023 | 37/278 | 1.08 (0.72-1.64) | 0.701 | 0.954 | 102/857 | 58/444 | 1.05 (0.73-1.52) | 0.779 | 0.935 | 96/804 | 64/497 | 1.09 (0.76-1.55) | 0.644 | 0.978 |
| SCC | 733/879 | 431/422 | 1.17 (0.98-1.40) | 0.077 |  | 899/1,023 | 265/278 | 1.05 (0.86-1.29) | 0.623 |  | 724/857 | 440/444 | 1.16 (0.98-1.39) | 0.086 |  | 702/804 | 462/497 | 1.04 (0.88-1.23) | 0.665 |  |
| Non-squamous | 108/879 | 49/422 | 0.82 (0.56-1.20) | 0.317 |  | 127/1,023 | 30/278 | 0.87 (0.57-1.35) | 0.540 |  | 99/857 | 58/444 | 1.09 (0.76-1.56) | 0.653 |  | 96/804 | 61/497 | 0.99 (0.69-1.42) | 0.964 |  |
| FIGO stage |  |  |  |  |  |  |  |  |  |  |  |  |  |  |  |  |  |  |  |  |
| I | 453/879 | 238/422 | 1.03 (0.84-1.26) | 0.795 | 0.320 | 539/1,023 | 152/278 | 1.00 (0.79-1.27) | 0.995 | 0.734 | 430/857 | 261/444 | 1.14 (0.93-1.39) | 0.213 | 0.946 | 434/804 | 257/497 | 0.94 (0.77-1.15) | 0.525 | 0.111 |
| II | 311/879 | 192/422 | 1.25 (0.99-1.57) | 0.058 |  | 389/1,023 | 114/278 | 1.08 (0.83-1.40) | 0.580 |  | 316/857 | 187/444 | 1.16 (0.93-1.46) | 0.187 |  | 283/804 | 220/497 | 1.26 (1.01-1.57) | **0.043** |  |
| III~IV | 27/879 | 21/422 | 1.835 (0.94-3.57) | 0.074 |  | 35/1,023 | 13/278 | 1.28 (0.60-2.72) | 0.529 |  | 29/857 | 19/444 | 1.12 (0.56-2.21) | 0.750 |  | 32/804 | 16/497 | 0.79 (0.39-1.62) | 0.523 |  |
| Tumor size, cm |  |  |  |  |  |  |  |  |  |  |  |  |  |  |  |  |  |  |  |  |
| < 4 | 580/879 | 328/422 | 1.13 (0.94-1.36) | 0.206 | 0.696 | 707/1,023 | 201/278 | 1.05 (0.85-1.30) | 0.649 | 0.696 | 558/857 | 350/444 | 1.21 (1.01-1.46) | **0.044** | 0.439 | 559/804 | 349/497 | 1.00 (0.83-1.20) | 0.994 | 0.489 |
| ≥ 4 | 294/879 | 157/422 | 1.04 (0.82-1.32) | 0.730 |  | 346/1,023 | 105/278 | 1.02 (0.78-1.34) | 0.872 |  | 289/857 | 162/444 | 1.02 (0.80-1.29) | 0.897 |  | 267/804 | 184/497 | 1.12 (0.89-1.41) | 0.342 |  |
| Pelvic LN |  |  |  |  |  |  |  |  |  |  |  |  |  |  |  |  |  |  |  |  |
| Negative | 703/879 | 388/422 | 1.11 (0.93-1.33) | 0.235 | 0.800 | 850/1,023 | 241/278 | 1.05 (0.86-1.29) | 0.652 | 0.502 | 687/857 | 404/444 | 1.14 (0.96-1.36) | 0.142 | 0.256 | 657/804 | 434/497 | 1.06 (0.89-1.26) | 0.518 | 0.518 |
| Positive | 202/879 | 116/422 | 1.10 (0.83-1.44) | 0.514 |  | 241/1,023 | 77/278 | 1.07 (0.78-1.45) | 0.688 |  | 187/857 | 131/444 | 1.25 (0.96-1.63) | 0.102 |  | 199/804 | 119/497 | 0.95 (0.73-1.25) | 0.715 |  |
| LVSI |  |  |  |  |  |  |  |  |  |  |  |  |  |  |  |  |  |  |  |  |
| Negative | 530/879 | 309/422 | 1.14 (0.94-1.38) | 0.189 | 0.671 | 649/1,023 | 190/278 | 1.05 (0.84-1.30) | 0.696 | 0.782 | 516/857 | 323/444 | 1.19 (0.98-1.44) | 0.075 | 0.891 | 503/804 | 336/497 | 1.06 (0.88-1.27) | 0.560 | 0.710 |
| Positive | 267/879 | 146/422 | 1.10 (0.86-1.41) | 0.451 |  | 316/1,023 | 97/278 | 1.09 (0.82-1.44) | 0.550 |  | 252/857 | 161/444 | 1.18 (0.93-1.50) | 0.183 |  | 253/804 | 160/497 | 1.03 (0.81-1.31) | 0.796 |  |
| Depth of cervical stromal invasion | | |  |  |  |  |  |  |  |  |  |  |  |  |  |  |  |  |  |  |
| ≤ 1/2 | 419/879 | 246/422 | 1.19 (0.97-1.46) | 0.101 | 0.654 | 513/1,023 | 152/278 | 1.08 (0.85-1.37) | 0.517 | 0.756 | 407/857 | 258/444 | 1.23 (1.00-1.50) | 0.050 | 0.625 | 401/804 | 264/497 | 1.05 (0.86-1.29) | 0.608 | 0.733 |
| > 1/2 | 466/879 | 257/422 | 1.09 (0.89-1.33) | 0.427 |  | 564/1,023 | 159/278 | 1.00 (0.79-1.26) | 0.992 |  | 454/857 | 269/444 | 1.11 (0.91-1.35) | 0.323 |  | 444/804 | 279/497 | 1.01 (0.83-1.23) | 0.956 |  |
| ER-expression |  |  |  |  |  |  |  |  |  |  |  |  |  |  |  |  |  |  |  |  |
| Negative | 482/879 | 263/422 | 1.11 (0.91-1.35) | 0.307 | 0.922 | 568/1,023 | 177/278 | 1.12 (0.90-1.41) | 0.309 | 0.745 | 462/857 | 283/444 | 1.17 (0.96-1.43) | 0.113 | 0.074 | 456/804 | 289/497 | 1.01 (0.83-1.23) | 0.910 | 0.782 |
| Positive | 41/879 | 23/422 | 0.95 (0.54-1.67) | 0.860 |  | 50/1,023 | 14/278 | 0.98 (0.52-1.85) | 0.948 |  | 32/857 | 32/444 | 1.65 (0.97-2.80) | 0.063 |  | 38/804 | 26/497 | 1.04 (0.61-1.78) | 0.893 |  |
| PR-expression |  |  |  |  |  |  |  |  |  |  |  |  |  |  |  |  |  |  |  |  |
| Negative | 505/879 | 278/422 | 1.11 (0.91-1.35) | 0.315 | 0.626 | 596/1,023 | 187/278 | 1.12 (0.90-1.40) | 0.305 | 0.328 | 475/857 | 308/444 | 1.23 (1.01-1.49) | 0.037 | 0.215 | 480/804 | 303/497 | 1.00 (0.82-1.21) | 0.982 | 0.454 |
| Positive | 18/879 | 8/422 | 0.72 (0.28-1.83) | 0.486 |  | 22/1,023 | 4/278 | 0.74 (0.25-2.20) | 0.586 |  | 19/857 | 7/444 | 0.52 (0.19-1.41) | 0.196 |  | 14/804 | 12/497 | 1.48 (0.65-3.39) | 0.355 |  |
| BMI, body mass index; FIGO, International Federation of Gynecology and Obstetrics; CIN, cervical intraepithelial neoplasia; SCC, squamous cell carcinoma; LN, Lymph Node; LVSI, lymph-vascular space invasion.  ^a^ Obtained in logistic regression models with adjustment for age, age at primiparity, menopausal status and BMI;  ^b^ According to the current WHO recommendations;  ^hom^ Homogeneity test;  The results were in bold, if *P* < 0.05. | | | | | | | | | | | | | | | | | | | | |
